# Progress in Medicine

**Published:** 1894-09-01

**Authors:** 


### PROGRESS IN MEDICINE.

### RENAL MEDICINE.

**Nephritic Colic.**—With reference to the old belief that cider has a beneficial effect on gravel, Dr. Blanc[1] has tested the action of valerianate of amyl, one of its constituents. In 2½ grain capsules every half hour up to five or six doses it is valuable for restraining the pain of nephritic colic and relieving the cystitis, though it has no influence on the calculus. In hepatic colic it gives great relief, and has the power of dissolving cholesterin. Its anæsthetic properties have been tested in various painful affections. Aussilleux speaks of the undoubted value of olive oil[2] in nephritic colic. Its action is apparently reflex, and more lasting than that of morphine. He prefers to give one or two ounces at a time, repeated as required. Contrexéville water[3] in the colic of oxaluria is discussed by Boursier, who gives notes of sixty-six cases. More than 2½ litres a day is not recommended. He found oxalate crystals in 150 cases out of 450 patients suffering from gravel. Hæmaturia and frequent painful micturition, with nervous troubles and dyspepsia, are usual concomitants of oxaluria. The pains caused by the gravel are more violent than those caused by uric acid.

Another remedy for nephritic colic is piperazene,[4] recommended by Dr. Blanc in doses of 7 to 15 grains twice a day fasting, dissolved in a carbonated water. Some patients after a few days have a return of the pain, followed by an expulsion of the stone, which has been diminished by the solvent action of the drug. However, its results are uncertain, and it should not be used hypodermically.

**Albuminuric Ulceration of the Bowels**[5] was discussed at the Medico-Chirurgical Society, when Dr. Dickinson brought forward twenty-two instances. The ulcers are not confined to one part of the bowel, nor to any special glands. They are frequently associated with hæmorrhage, and eight other cases of extravasation without ulcers were mentioned. Peritonitis and sometimes perforation might follow. The majority of the cases were those of granular kidney. Hale White gave similar instances, and believed that the lesions were the result of ulcerative colitis, rather than of hæmorrhage. The discussion is of interest when compared with Herter's theory of uramia, recently mentioned in these columns.

**Nucleo-albumen** is sometimes found in the urine, in addition to other proteid substances, when damage is done to the tissue cells, as by limiting the supply of oxygen. Temporary compression of a large vessel will sometimes, according to Pichler and Vogt, suffice to cause it. Cyclical albuminuria is often of the nucleo variety, and may imply injury to either the renal tissue or to other parts of the organism. The substance is soluble in acetic acid, but precipitated by magnesium sulphate.[6] F. Vivenza shows that there is loss of hœmaglobin from the blood in passing through the kidneys, which is proportional to the water eliminated, and is associated with the formation of urinary pigment. This loss is greater than can be accounted for by the destruction of red corpuscles, which normally occurs during the passage of the blood through the kidney.

Millard's trichloracetic acid test has been highly spoken of because it does not precipitate peptones. The peptone usually found, however, is usually a proteose or albumose, and these substances are partially precipitated by it in the cold, though the precipitate dissolves on warming. Landon C. Gray holds that many functional nerve diseases are very frequently accompanied by albuminuria, excess of uric acid, and oxalates as a result not a cause of the disease. They are but little benefitted by diet, but relief of the nerve symptoms is followed by disappearance of the kidney disease. It has been pointed out that these results are not confirmed by other observers, and that Millard's test, which he employs, may have a less serious significance than the ordinary ones. Alterations of blood pressure, as in epileptic fits, are frequently followed by albuminuria. Paul Tiemann gives an elaborate summary of the literature of the albuminuria of adolescence.[10] In 2,000 examinations he found 9 per cent. of cases of albumen, and 27 per cent. in young male subjects. In a later series he found that out of 240 boys 22 per cent. showed the reaction for albumen.

W. F. Fenton shows that great discrepancy exists between clinical symptoms and the morbid anatomy of the kidneys.[17] Thus the senile changes in the kidney may be very great without albumen or increase of urine. Similar changes occur in fevers without albuminuria. In diphtheria he found degeneration of the tubal epithelium, even where there was only slight albumen. In certain non-febrile or toxœmic cases he finds structural changes without renal symptoms, while in acute nephritis there is actually less cloudy swelling of renal cells than in many toxœmic cases with little albuminuria. The atrophy of the kidney, accompanied by arterio-sclerosis, is very marked in old age without any clinical symptoms whatever.

Dr. T. Harris has described[12] a method of preserving urinary casts and other organic deposits. After settling for twelve hours the deposit is drawn off with a pipette and dropped into a tube nearly full of preservative fluid. The lower end is drawn out to a point and has a hole one-sixteenth of an inch in diameter. The upper end is closed by a rubber cork, and the deposit sinks in about twelve hours to the bottom, whence a drop can be driven out by pressure on the cork, for mounting on a slide. The fluid he uses is Pot. acetat. grm. 60, chloroform 10 c.c., aq. destillat. litre 1. Some specimens were preserved two years. Gurrieri gives a needful warning that a little chloride of sodium should be added to Spiegler's test solution to avoid errors if that salt should happen to be absent from the urine.[13]

**Limewater** given in the food may cause an ammonia odour in the urine.[14] Abel, of the Johns Hopkins' Medical School, has made researches on the subject, and finds that calcium carbamate is formed. Carbamic acid, one of the precursors of urea, appears to combine with the lime in the system, and the organism avails itself of the easily soluble carbamate of calcium to eliminate any excess of lime. This salt easily gives off free ammonia on standing. This hitherto unknown reaction of the urine is important and interesting. Conti[15] contributes to our knowledge of acetonuria, that in infective diseases acetone is generally present, but it has no relation to the toxicity of the urine; that it frequently appears after operations without any symptoms in the state of the patient, and it is not a necessary sequel to peritoneal operations or the use of sublimates; lastly, it occurs occasionally in normal individuals.

Köstlin[16] concludes that simple warm, salt, or mustard baths have no effects on the nitrogenous excretion, but that this is lessened by a bath of Stassfurter salt containing potash, magnesium, and calcium.

The relief given in severe renal asthma by venesection is exemplified by a case reported by R. Kull[17], who drew off 15 ounces. The patient could at once lie down, and all distressing symptoms had disappeared after a few hours. Uric acid to the amount of ·015 per cent. was found in the blood. A variety of Brown Sequard's renal extract has been tried in chronic Bright's disease. Two c.c. of an extract was injected once, and then twice daily.[18] There was no change in the quantity of urine or chlorides eliminated, but a slight increase of urea, greater toxicity of the urine, and a large increase of phosphates, and a general improvement in the patient's condition, according to Teisser and Frenkel.

Piccinini[18] states that peptone is to be found in the urine after the administration of guiacol, antefebrin, or salicylates, by precipitating with ammonium sulphate. Wunderlich,[19] reporting on 125 operation cases with regard to the effect of anæsthetics on the kidneys, says that an existing albuminuria is often increased by etherisation (chloroform was not tried in these cases), it can be caused in healthy persons by chloroform or ether, more frequently by chloroform. Casts may appear after an anæsthetic, most often after chloroform, and are increased if previously present. The albumen was slight in amount and disappeared in a few days.

In the eclampsia of pregnancy chloroform and ether are often employed as remedial agents, but R. C. M. Page draws attention to the danger of them both, and warmly advocates the treatment[20] of uræmic cases generally by opium and veratum viride combined. He frequently gives a hypodermic injection of morphia, and then 5 to 10 m. tinct. of veratrum by the same syringe. He claims that the latter drug is practically safe if given with opiates, and that it acts quickly and more efficiently than any other remedy.

**The Bacteria of Pyelonephritis and Cystites,** according to Sternberg, are usually introduced through the urethra by catheterisation or injections. The most frequent is the bacillus coli communis, which is constantly present about the anus and probably under the prepuce. There is no evidence that the bladder is reached by the direct invasion of bacteria without mechanical assistance, nor does the B. coli exist in the normal urethra.[21]

**The Relative Proportions of the two Proteids** in the urine in cases of albuminuria have been investigated by Francis D. Boyd.[22] He concludes that (1) in albuminuria both the proteids of the blood and serum are usually present, but this is not true in every case. (2) We cannot diagnose the form of lesion from the proportions of the two proteids. (3) The proportions of serum albumin and globulin may vary widely. (4) Even in amyloid degeneration the globulin may not be in excess. (5) In the albuminuria of pregnancy both are present, the globulin usually being in excess. (6) In heart disease without chronic kidney lesions globulin is generally more than in chronic intestitial nephritis. (7) In acute nephritis with hæmaturia the proportions are about equal, but globulin may be increased if there is blood in the urine.

At the N. Y. Pathological Society[23] another instance of extensive cystic degeneration was shown. The kidneys weighed nine ounces each and the remaining tissue was affected with chronic parenchymatous nephritis. Two explanations of the condition had been given, either that it was an excessive development of the small cysts seen in the cirrhotic kidney, or that these cysts represent the low degree of fœtal cystic kidney.

**Intermittent Hydronephrosis** is attributed by Tuffier to the presence of a moveable kidney.[24] It is nearly always found in women and connected with the right kidney. It had been obtained experimentally in animals. The intermittence may be caused by the bend in the ureter getting straightened out by the mobility of the kidney, or in other cases the tension of the kidney overcomes the impediment and forces out the urine. He advises the operation of fixing the kidney.

[1] Amer. J. Med. Sc., March, 1894. [2] Ther. Gaz., March, 1894. [3] B.M.J., June 23rd, 1894. [4] Amer. J. Med. Sc., April, 1894. [5] N. Y. Med. Record, Feb. 17th, 1894. [6] Ep. B.M.J., June 2nd, 1894. [7] Glas. Med. J., May, 1894. [8] B.M.J., Jan. 27th, 1894. [9] Med. Week., May 4th, 1894. [10] N. Y. Med. J., March 27th, 1894. [11] Practitioner, May, 1894. [12] B.M.J., June 23rd, 1894. [13] Med. Record, April 13th, 1894. [14] N.Y. Med. J., May 12th, 1894. [15] B.M.J., May 19th, 1894. [16] Amer. J. Med. Sc., Jan., 1894. [17] B.M.J., June 2nd, 1894. [18] B.M.J., May 12th, 1894. [19] Annals of Surg., May, 1894. [20] N. Y. Med. J., May 12th, 1894. [21] Amer. J., Med. Sc., June, 1894. [22] Ed. Med. J., May, 1894. [23] Med. Record, March 31st, 1894. [24] Med. Record, April 28th, 1894.

